# St. John’s Wort (*Hypericum perforatum*) Products – How Variable Is the Primary Material?

**DOI:** 10.3389/fpls.2018.01973

**Published:** 2019-01-24

**Authors:** Francesca Scotti, Katja Löbel, Anthony Booker, Michael Heinrich

**Affiliations:** ^1^Pharmacognosy and Phytotherapy Group, Pharmaceutical and Biological Chemistry, UCL School of Pharmacy, London, United Kingdom; ^2^Division of Herbal and East Asian Medicine, Department of Life Sciences, University of Westminster, London, United Kingdom

**Keywords:** avicularin/guaiaverin, *Hypericum perforatum* (Saint John’s wort, SJW), materia prima, natural variability, quality control, subspecies boundaries, value chains

## Abstract

**Background:** Saint John’s wort (*Hypericum perforatum* L., HP) is commonly registered in Europe under the THR scheme (Traditional Herbal Registration) or licensed as a medicine. Nonetheless unregulated medical products and food supplements are accessible through the internet which are often of poor quality. The species’ natural distribution stretches through large regions of Europe to China and four subspecies have been distinguished. When compared to the European Pharmacopoeia reference, the presence of additional compounds was linked to so-called Chinese HP.

**Aim:** In order to obtain an integrated picture of the entire chemoprofile, the chemical composition of HP *materia prima* was studied using a combination of techniques well-established in the relevant industries. The impact of phytogeographic factors on the *materia prima* can shed light on whether the variability of the final products is strongly influenced by these factors of whether they relate to poor processing, adulteration, or other factors linked to the processing of the material.

**Methods:** Eighty-six *Hypericum* samples (77 *H*. *perforatum*) were collected from 14 countries. Most were authenticated and harvested in the wild; others came as roughly ground material from commercial cultivations, markets and pharmacies. The samples were analyzed using HPTLC and ^1^H-NMR-based principal component analysis (PCA).

**Results and Discussion:** Limited chemical variability was found. Nonetheless, the typical fingerprint of Chinese HP was observed in each specimen from China. Additional compounds were also detected in some samples collected in Spain. Rutin is not necessarily present in the crude material. The variability previously found in the marketed products can be ascribed only partially to the geographical origin of harvested material, but mainly to the plant part harvested, closely related to harvesting techniques, processing and probably time of harvest.

**Conclusion:** HP can be sourced in a consistent composition (and thus quality) from different geographical sources. However, chemical variability needs to be accounted for when evaluating what is considered authentic good material. Therefore, the processing and good practice are all stages of primary importance, calling for a better (self-)regulation and quality assurance along the value chain of an herbal medical product or botanical.

## Introduction

Saint John’s wort (*Hypericum perforatum* L. – HP, Hypericaceae) has been used traditionally across Europe for centuries and in contemporary society it plays an important medical role. Its renown ability to treat wounds is being investigated (Oztürk et al., 2007; Süntar et al., 2010, 2011) but, most importantly, it is now widely used as a prescription or over the counter medicine to treat minor to moderate depression (licensed medicines) or ‘low mood’ (registered products). It was found that its activity is comparable to antidepressants when dealing with mild to moderate depression (Apaydin et al., 2016). In general, it is a licensed drug in many European countries and in the United Kingdom it is registered under the THR scheme. While considerable effort has gone into understanding the chemistry and pharmacology of commercially used materials, little attention has been paid to the biological and chemical complexity of the starting material and specifically to the diversity within the taxon *H. perforatum* (Dauncey et al., 2017). HP could be considered as an umbrella term for different *Hypericum* taxa united by the use as herbal medicines esp. in ‘mood disorders.’

The species used medicinally in more recent Western medicine is *Hypericum perforatum*. HP is native to Eurasia, it is found in Europe (excluding the extreme north), the “Levant and western Saudi Arabia to NW India (Uttar Pradesh), Transcaucasia, Turkmenistan to Altai, Angara-Sayan and NW Mongolia; China (W. Xinjiang and from Gansu east to Hebei, south to Jiangxi and west to Yunnan)” (Robson, 2002). It is also found in NW Africa, including Canary Islands, Madeira and Azores. It has been introduced in the American continent, where it can be found from Canada to Argentina, in the Republic of Sudan (Jebel Marra), South Africa, Reunion, Australia, New Zealand, and Japan.

Robson (2002) proposed the distinction of four subspecies, based on minor morphological traits and with a well-defined geographical distribution of two of these subspecies, while the two Central/Western subspecies overlap through a large range of their territories (Dauncey et al., 2017).

Apomictic reproduction is known to give surge to a number of interfertile hybrids, morphologically different but within a continuum, therefore rendering taxonomic identification extremely difficult (Dickinson, 1998; Bicknell and Koltunow, 2004). This tendency, through time, has led to a considerable variability among the morphology of *H. perforatum* species. The state of current knowledge (Robson, 2002) is that these four subspecies possibly originated from a common ancestor (Western Siberia) which, interbred with other *Hypericum* species, gave birth to morphologically distinct, but recurring and geographically restricted, hybrids that are now being recognized as subspecies. According to Robson (2002), from the common ancestor first ssp. *songaricum* and ssp. *perforatum* evolved. Subsequently, ssp. *veronense* and ssp. *chinense*, respectively, evolved. According to Robson ssp. *songaricum* is the closest to the original ancestor. Ssp. *chinense* is seen as particularly distinct from its predecessor, and further away evolutionarily.

This situation raises two distinct but interrelated questions:

(1) What is the quality of the material currently available in different markets and how is this linked to the production of the *materia prima* and the subsequent value chains (Booker et al., 2018).
(2) How does the intrinsic variability of *H. perforatum* impact on the composition of the products available?

Previous investigations have revealed how the quality of food supplements (botanicals) is variable (Zheng and Navarro, 2015; Booker et al., 2016a,b; Barrella et al., 2017; Ruhsam and Hollingsworth, 2018), and studies conducted on the chemical quality of HP products showed problems specific to this species, (Frommenwiler et al., 2016; Booker et al., 2018), including strength and dosage inconsistencies and the presence of food dyes. A chemical pattern previously identified by Huck-Pezzei et al. (2013) and Frommenwiler et al. (2016) was initially labeled “Chinese HP” as it was almost only found in commercial products of Chinese origin. The Chinese material, and, therefore, the ssp. *chinense* has been thought to constitute a specific chemotype differing from the other subspecies. The previously found Chinese HP fingerprint (Frommenwiler et al., 2016; Booker et al., 2018) showcased three main features different from the EP and USP HPTLC standards and raw herbal material analyzed in those studies:

(1) Absence of yellow fluorescent zone at Rf = 0.18, under the blue zone of chlorogenic acid.
(2) Lower intensities of the bands found in the lower third of the chromatogram.
(3) Additional fluorescent band at Rf = 0.49.

As a consequence, it was thought expedient to analyze HP crude drug material collected across the world and evaluate the chemical profiles via nuclear magnetic resonance (NMR) spectroscopy and high performance thin layer chromatography (HPTLC) to verify whether this profile can only be found among Chinese specimens and whether other chemical variation exists in the naturally occurring crude drug material around the globe.

## Aims and Objectives

(1) To systematically compare the variability of HP samples originating from diverse geographical locations covering both the main natural range of HP and some selected regions of agricultural production.
(2) To assess what constitutes a good *materia prima*, inclusive of the biodiversity and variability.
(3) To assess whether all accessible subspecies/geographical sources constitute good starting material and to guarantee a good final product.
(4) To find an appropriate method for the evaluation of the *materia prima*.

This has been achieved by analyzing samples from different countries across the world and comparing the data with those provided by the previous study conducted on the finished products available on the market (Booker et al., 2018).

## Materials and Methods

All solvents were purchased from Merck KGaA, Fisher Scientific Ltd. and VWR International LLC, except of deuterated methanol which was purchased from Cambridge Isotope Laboratories Inc.

### Sample Collection

Eighty-six samples (for a detailed description see the Supplementary Material) were collected and dried at room temperature and voucher specimens for the unprocessed samples are deposited at UCL School of Pharmacy Herbarium.

Specimens were harvested in the wild or obtained from commercial cultivations, the flowering aerial parts were collected (unless differently stated in the Supplementary Material) and dried in the shade.

Commercial processed samples were purchased or donated in the form of roughly ground plant material.

### Reference Standards

St. John’s wort dry extract (European Pharmacopoeia, EP, Reference Standard 01131, code: Y0001050, batch: 2.0), avicularin and guaiaverin were obtained from Sigma-Aldrich Inc. while rutin (L10815B002) from Adooq Bioscience. Hypericin primary reference standard (batch HWI 01814-1) was purchased from HWI ANALITIK GmbH.

### ^1^H-NMR Spectroscopy

Nuclear Magnetic Resonance analysis was carried out using a Bruker Avance Spectrometer featuring a QNP multi-nuclear probe head with z-gradient/5 mm cryoprobe head operating at 500.13 MHz. Spectra were acquired at 298 K, using 64k data points, line broadening factor = 0.16 Hz, pulse width = 30°, relaxation delay d1 = 1 s. Each run was subjected to 256 scans. The acquired data was processed using TopSpin 3.2 software. Chemical shifts were calibrated to the tetramethylsilane (TMS) signal.

#### Sample Preparation for NMR

The dried material was ground finely using a blender. The solvent choice and the analytical methods followed the outline of the previous study on HP marketed products (Booker et al., 2018) for the purpose of comparability.

Fifty milligrams of powder was extracted in 1 mL deuterated methanol, vortexed for 20 s, sonicated for 5 min at room temperature and finally centrifuged for 5 min at 13,000 rpm. 0.6 mL of supernatant was sent for analysis.

Reference standard pure compounds were simply dissolved in methanol, at the concentration of 1 mg/mL and 0.6 mL was analyzed.

### Principal Component Analysis of Data

^1^H-NMR signals were calibrated to the TMS peak. The spectra acquired were converted to ASCII file using AMIX 3.9.14. Using only positive intensities and no scaling, buckets of 0.04 ppm were created using the multivariate analysis software. Via the use of Excel, the NMR elaborated data was introduced onto SIMCA 14.0, the software utilized for the principal component analysis (PCA). Sample 22 was analyzed twice and, after different trials, it was established that no scaling in SIMCA gave a statistical model that was to be considered more reliable based on the proximity of the sample 22 repeats in the plot.

### HPTLC

HPTLC was performed using a CAMAG setup consisting of a Linomat 5 semi-automated sampler, automatic developing chamber 2 (ADC2), TLC plate heater III and TLC visualizer coupled to visionCATS 2.1 software. The HPTLC plates Silica gel 60 F_254_ used for stationary phase were purchased from Merck KGaA (Darmstadt, Germany).

#### Sample Preparation for HPTLC Analysis

Five hundred milligrams of powdered material was extracted with 5 mL of methanol, shaken on a rotary mixer for 20 s, sonicated 10 min in at 60°C and filtered using Millex^®^ Syringe filter unit 0.45 μm.

The references hypericin and quercetin were dissolved in methanol, while rutin in acetone, with a concentration of 1 mg/mL then sonicated for 10 min at 60°C. Rutin had to be filtered through a Millex^®^ syringe filter unit 0.45 μm, to remove any residual suspended particle prior to use. The EP standard was prepared in methanol at a concentration of 100 mg/mL.

#### HPTLC Analysis

The method used reflects the one published by the HPTLC association for the extraction and analysis of HP powdered drug (HPTLC, 2016). Each plate was visualized under white light and UV 254 nm, prior to sample application in order to later correct for the background. 2 μL of sample and standards, were spotted on the plates in bands of 8 mm. The plate was developed in the automatic developing chamber at 33% humidity, with 20 min saturation time, 10 min activation time and 5 min pre-drying. The mobile phase consisted of a freshly prepared mixture of ethyl acetate, dichloromethane, HPLC-grade water, formic acid, glacial acetic acid in the proportion 100:25:11:10:10 (*v/v/v/v/v*). After development, the plate was visualized under white light, UV 254 and 366 nm. Prior to derivatization the plate was heated at 100°C for 3 min and subsequently dipped, while still hot, in NP reagent first (1 g 2-aminoethyl diphenylborinate in 200 mL ethyl acetate) and then PEG reagent (10 g polyethylene glycol 400 in 200 mL dichloromethane), for the detection of flavonoids. The plate was then visualized under white light and UV 366 nm.

## Results and Discussion

**FIGURE 1:**
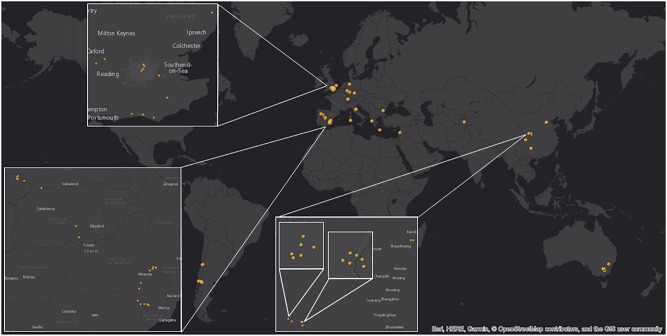
The *H. perforatum* samples used in this project have been collected in 14 countries.

With the intention to define the chemical profile of HP, the project embarked on the analysis of samples trying to identify the common as well as the variable chemical components of HPs from different geographical regions. Therefore, our collection of 77 HP samples from 14 different countries covered native Europe extensively (South England, Portugal, Spain, Germany, Switzerland, Italy, Bulgaria, Greece), Lebanon, Tajikistan, China and areas of introduction such as South America (Chile, Argentina) and Australia (Figure 1).

As a first step, the chemical composition of different sections of the aerial parts, the traditionally recommended drug, were analyzed. One single HP specimen from Southern England (nr 53) was cut in 4 parts (sample 53#1 0–18 cm, lower; 53#2 18–37, cm lower intermediate; 53#3 37–54 cm, upper intermediate; 53#4 54–65 cm, flowering tops); in addition, samples containing only leaves (sample 53#5) and only flowers (sample 53#6) were taken from the same specimen.

**FIGURE 2:**
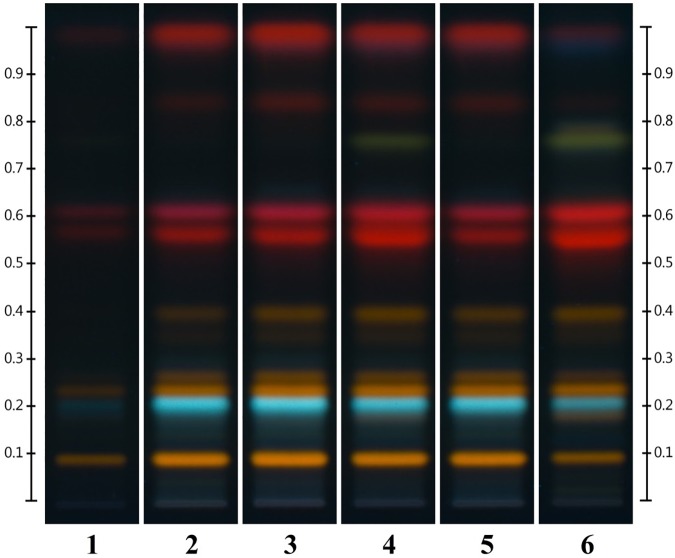
Different parts of *Hyperici herba* (from sample 53) analyzed by HPTLC. Tracks: (1) lower part (0–18 cm); (2) lower intermediate part (18–37 cm); (3) upper intermediate part (37–54 cm); (4) flowering tops (54–65 cm); (5) leaves only; (6) flowers only.

HPTLC analysis showed, as expected, a variation in the chemical content between parts (Figure 2). Material derived to the lower section of the aerial parts was constituted only of woody stems and the methanolic solution obtained was light yellow. The chromatographic fingerprint showed very low levels of detectable components. Samples 53#2 and #3 both contained leaves and slimmer woody stems, the methanolic solution obtained was dark brown in color and the HPTLC fingerprint seemed perfectly acceptable for an HP product.

Sample #4 represented the flowering tops, the part to harvest based on the pharmacopeial requirements (“Whole or fragmented, dried flowering tops of *Hypericum perforatum* L., harvested during flowering time” BP 2018, Ph. Eur. 9.3 Update). In this case the methanolic solution is dark red and the fingerprint is similar to the previous two samples with the addition of a green band at Rf = 0.77 and slightly more concentrated bands of hypericin derivatives (red bands between Rf = 0.54 and Rf = 0.63). As expected, the sample exclusively made of leaves (53 #5) has exactly the same fingerprint of #2 and #3 but the methanolic solution is green in color. Finally, the flower sample, 53 #6, shows a fingerprint with a level of hypericins comparable to #4, the green band at Rf = 0.77, a faint yellow band right above said green band and a much fainter top elution band.

“Chinese HP” with its specific fingerprint characteristics could be adulterated with other species. Therefore, nine samples of other *Hypericum* species growing in China were collected and analyzed by HPTLC including *H. ascyron* (F6), *H. acmosepalum* (F7, 9), *H. uralum* (F8), *H. densiflorum* (F10), *H. beanii* (F11), *H. patulum* (F12), *H. japonicum* (F14), *H. elodeoides* (F15).

**FIGURE 3:**
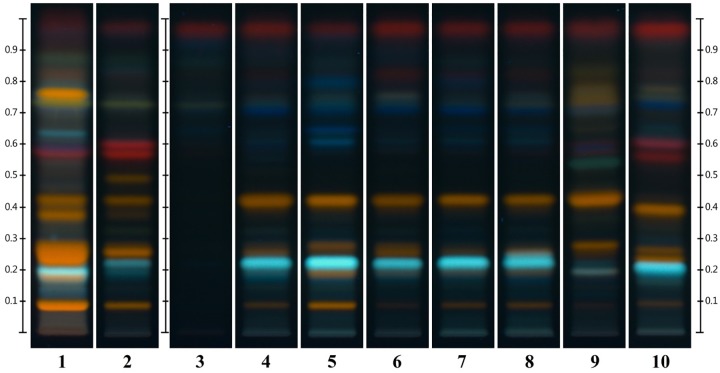
Similarities and differences between EP standard for HP (track 1), Chinese HP (track 2) and other species of *Hypericum: H. ascyron* (track 3), *H. acmosepalum* (track 4), *H. uralum* (track 5), *H. densiflorum* (track 6), *H. beanii* (track 7), *H. patulum* (track 8), *H. japonicum* (track 9), *and H. elodeoides* (track 10).

The HPTLC (Figure 3) and NMR (Figure 11) results clearly show that none of the fingerprints features the yellow band at Rf = 0.49 (present in the Chinese *H. perforatum*, Figure 3, track 2). The other *Hypericum* species’ fingerprints (Figure 3, tracks 3–10) are very distinct from *H. perforatum*’s. Except for *H. elodeoides* (Figure 3, track 10), they do not contain hypericins and it is unlikely that they could have been added, accidentally or on purpose to boost the products’ specifications.

**FIGURE 4:**
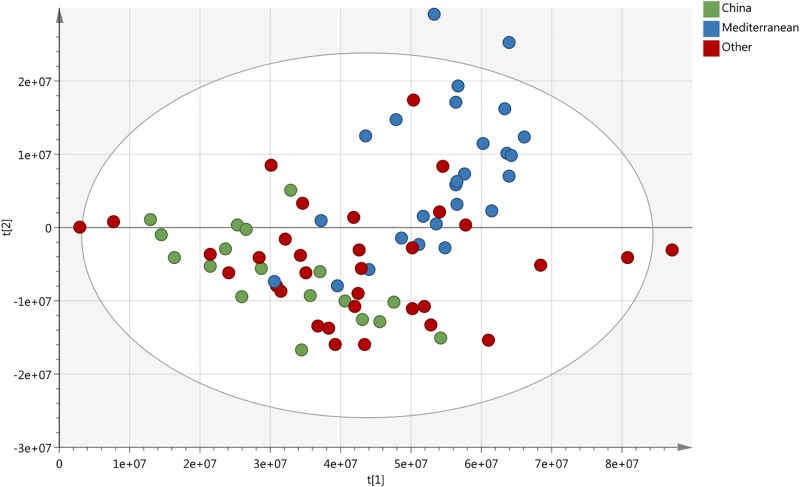
PCAscore plot of 77 samples of crude drug of HP coming from 14 different countries, highlighting Chinese samples (green), Mediterranean samples (blue), and all others (red).

Principal component analysis of NMR data relative to the HP crude drug samples altogether shows a fairly homogeneous spread, without any starkly prominent difference (Figure 4). Nonetheless the samples from China and those from the Mediterranean area form separate clusters. Western/Central European samples from Germany and England overlap over both clusters. Analysis of the flavonoid specific area 6–9 ppm failed to show any further difference (see Supplementary Material), instead showing an even more homogeneous distribution, with less similarities but without any type of clustering and a broader distribution.

HPTLC analysis of the samples highlighted a few main differences across the collection, namely the presence of the “Chinese HP” fingerprint, the separate presence of the extra yellow band (Rf = 0.49) in other samples, the presence/absence of rutin, low flavonoid concentrations and differences in the hypericins content.

**FIGURE 5:**
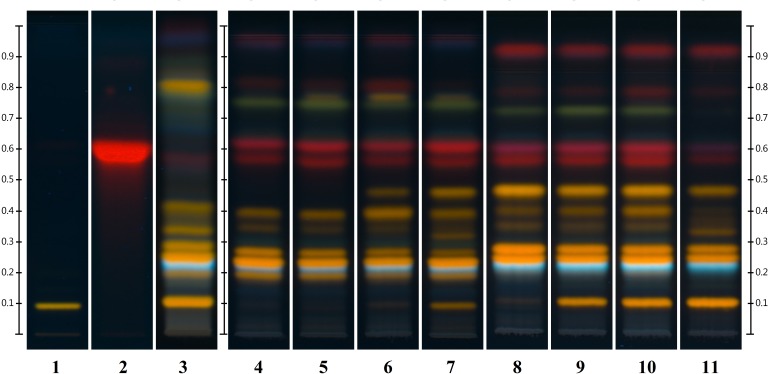
Comparison between Chinese and Spanish samples fingerprints; Tracks: (1) rutin standard; (2) hypericin standard; (3) EP reference standard for HP; (4–7) some samples from Spain (original sample nr 42–45); (8–11) some samples collected in China (original sample nr 59–61 and 64).

Each of the samples acquired from China showed the “Chinese HP” fingerprint, with, most notably, an extra compound, represented by the yellow band with Rf = 0.49 and the missing yellow band at Rf = 0.18 (Figure 5). This seems to define a specific chemotype for specimens belonging to the postulated ssp. *chinense* (also described as geographically restricted to China). Interestingly, a yellow band with Rf = 0.49 was also detected in 50% of the samples collected in Spain (8 out of 16, from two separate regions). In the latter cases though, the persistence of the yellow band with Rf = 0.18 indicates a fingerprint distinct from the Chinese one (Figure 5). The compound at Rf = 0.49 was otherwise not detected in any other sample of our collection.

Additionally, rutin is not necessarily found in the crude drug material as 38% (27 out of 71 samples with sufficient flavonoid concentration to be able to read rutin band) of the samples did not show the corresponding band; Chinese samples were always found to contain rutin, in different concentrations, while the majority (81%) of Spanish material does not contain it. On the other hand, all the marketed products analyzed by Booker et al. (2018) contain rutin.

HPTLC analysis highlighted the presence of lower concentrations of compounds in samples consisting of processed material (purchased or donated in the form of roughly chopped material). Samples 1–5, 22, 23, 24, 65, 66 and 86 were obtained from commercial sources (producers, pharmacies, markets), allegedly being simply roughly processed *materia prima*. Visual inspection revealed roughly chopped herbal material, making it difficult, if not impossible to determine the identity of the plant with the naked eye; in addition, most of them included a high amount of woody material (stems). In the HPTLC analysis samples 1–5 (purchased at herbal markets, in three different regions of China: Yunnan, Hebei, Shanxi) showed an extremely low content of the typical HP compounds at the concentration examined. Samples 22–24 (respectively, purchased as loose material in a pharmacy in Crete, Greece; in a pharmacy in Chile, and acquired through a manufacturer in Chile) and 65–66 (both samples acquired from a manufacturer’s cultivation in Bulgaria) show better concentration but still among the lowest across the whole selection. This could be due to the apparent higher amount of woody material present in the mixtures.

This observation could be linked to the results obtained from the HPTLC analysis of the different sections of the aerial parts. The fainter fingerprint of the processed material could be due to the harvesting practice cutting further down the stem and this including a higher percentage of the woody material. Given that the wood itself does contain extremely low quantities of flavonoid compounds, this constitutes a natural bulking agent from the same plant. Whether this was done intentionally or due to a lack of knowledge cannot be ascertained in this study. Of note, for products regulated as botanicals/food supplements, this would not constitute an adulteration, but for herbal medicines it would, if the regulation follows, for example, the European Pharmacopoeia.

Alternatively, the fainter fingerprint could be ascribed to purchasing material from middlemen, implying that little information is available to the processors as to when the material was harvested and handled. Time and conditions of storage can lead to the degradation and oxidation of components, and therefore a lowering of their concentrations.

Based on our analysis, NMR-based PCA is unable to pick up on the composition differences detected via HPTLC. Moreover, the contribution of a single compound on the overall NMR spectra is minimal, especially when considering complex spectra such as those obtained from total plant extracts.

Hypericins, namely hypericin and pseudohypericin, are easily spotted in HPTLC plates treated with NP/PEG as two close red bands at Rf = 0.55–0.60. Their concentration varies across the collection of samples examined, ranging from thick brilliant to faint dark bands. However, no systematic correlations with regions of origin could be demonstrated. These differences can sometimes be associated with overall low flavonoid content (as in the case of commercial samples), but at times they do not directly correlate. As previously explained their lower content can be due to a lower proportion of flowers and leaves in the samples, the age of the material (often unknown in the case of commercial samples) but can as well be explained by time of the day/season when the material was collected. The failure to find a distinguishable marker peak for hypericin in the NMR spectra reinforces the idea that the NMR-PCA plot would not have taken into consideration the hypericin content differences.

### Avicularin Versus Guaiaverin

As previously mentioned, samples of Chinese origin analyzed were found to have a peculiar fingerprint, characterized mainly by the presence of an extra compound at Rf = 0.49. It was initially identified, based on mass spectrometry, as avicularin, or quercetin-3-*O*-α-arabinofuranose, which had previously been isolated in HP (Wei et al., 2009). Another quercetin-glycoside, guaiaverin (quercetin-3-*O*-α-L-arabinopyranoside) though, with the same molecular weight and the same fragmentation pattern as avicularin was previously isolated from *H. maculatum* (Zheleva-Dimitrova et al., 2012), raising doubts relative to the identity of the yellow band at Rf = 0.49 (Booker et al., 2018).

**FIGURE 6:**
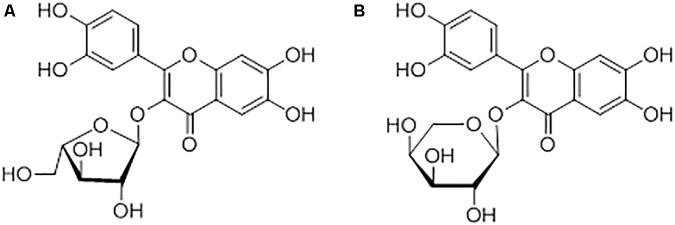
Structures of avicularin **(A)** and guaiaverin **(B)**.

**FIGURE 7:**
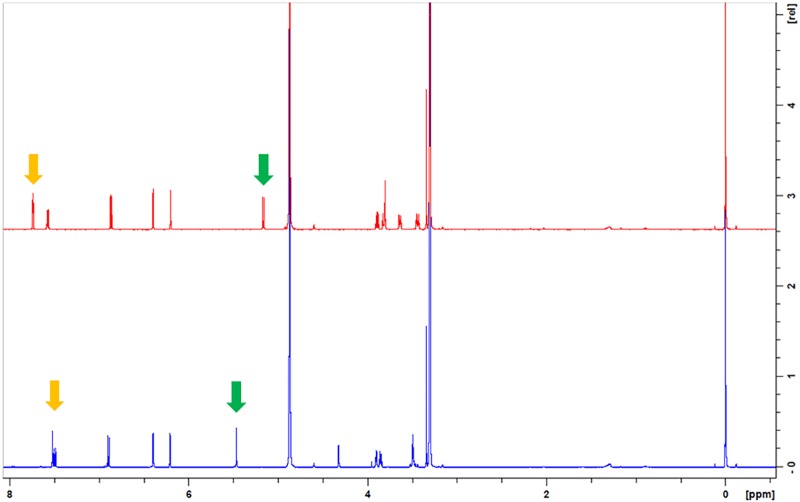
NMR spectra (500 MHz) of avicularin (blue) and guaiaverin (red) in CD_3_OD, highlighting particularly useful diagnostic features at 7.52 ppm/5.46 ppm (avicularin) and 7.74 ppm/5.16 ppm (guaiaverin).

**FIGURE 8:**
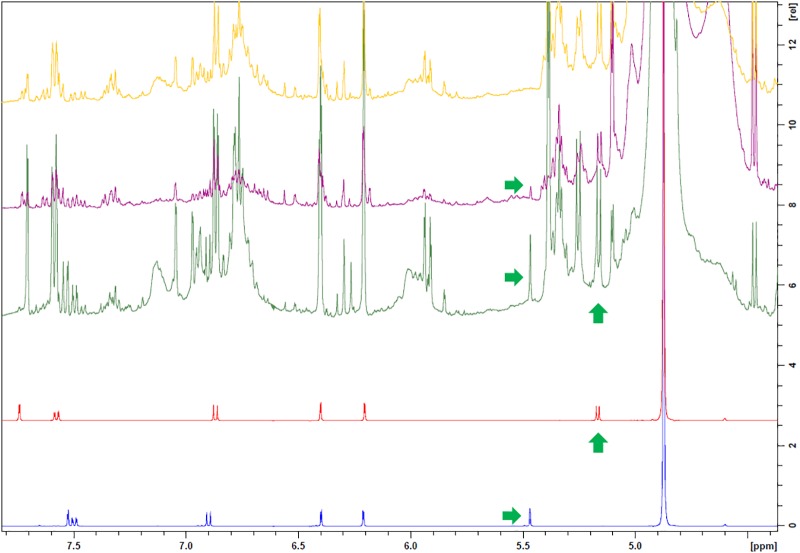
Spectra of avicularin (blue) and guaiaverin (red) compared to sample 59 (from China, green), 41 (from Spain, with extra band, purple) and 40 (from Spain, no extra band, yellow).

Their molecular structures are similar (Figure 6) but their NMR spectra differs and distinct signals can be identified (Figure 7). Pure compounds were compared to identify the samples’ NMR fingerprints. Peaks at δ (500 MHz, CD_3_OD) 5.47 (s) and 5.18 (d) ppm, found, respectively, in avicularin and guaiaverin, in an area of low signal crowding, provide a means for distinguishing between the two compounds and were chosen as marker signals. The singlet at 5.47 ppm is found in a representative sample of both Chinese and Spanish samples (No. 59 and 41, respectively), but is missing in another Spanish sample (Figure 8, sample 40, yellow) that did not show the extra band at Rf = 0.49. Therefore, the samples with the band at Rf = 0.49 are likely contain avicularin. The doublet at 5.18 ppm seems to be present in all samples’ spectra, but as it appears in an area of high signal crowding it did not seem appropriate to derive a clear conclusion solely based on NMR. Next, the possibility of both compounds being present (guaiaverin being present in a very low concentration) was investigated using HPTLC.

**FIGURE 9:**
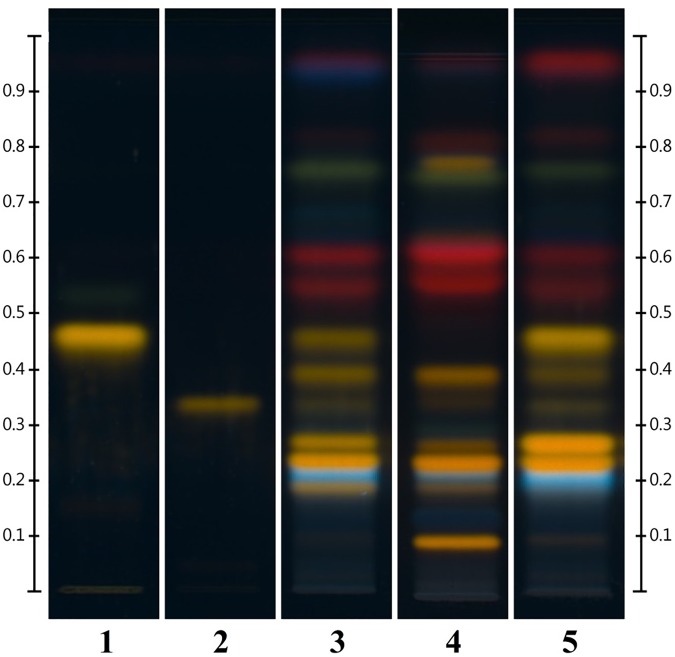
HPTLC detection (366 nm) of avicularin (track 1) and guaiaverin (track 2) in the crude drug material; tracks: (3) sample 41 from Spain; (4) sample 51 from Germany (lacking the extra component at Rf = 0.49); (5) sample 59 from China.

HPTLC analysis following the HP protocol showed a significant separation between avicularin and guaiaverin and based on this result the band at Rf = 0.49 represents avicularin (Figure 9) but not guaiaverin. Additionally, the band corresponding to guaiaverin (Rf = 0.30) was detected in both Chinese and Spanish samples, indicating the presence of a mixture of the two, with guaiaverin being present in a much lower concentration (Figure 9). Guaiaverin was detected also in other HP samples investigated (represented by German sample 51, in Figure 9).

### *Materia Prima* Versus Finished Products

**FIGURE 10:**
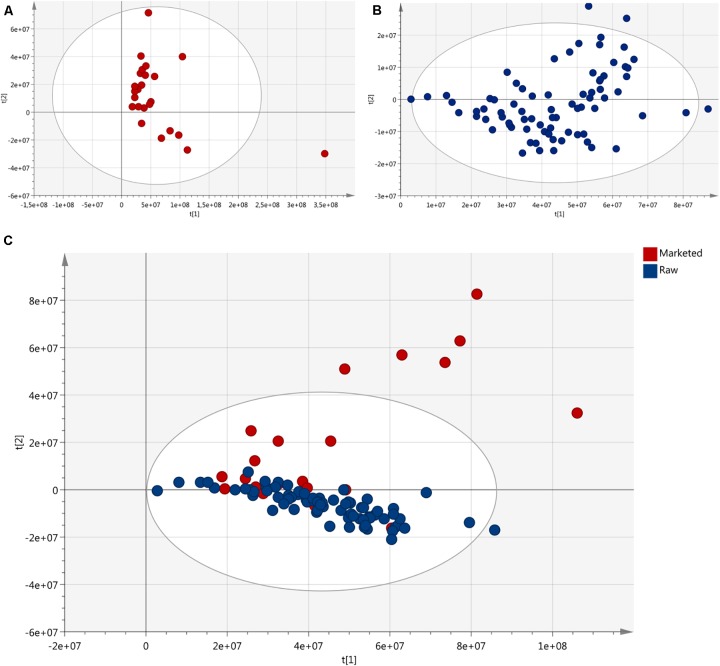
PCA score plots: **(A)** marketed products (no extracts) from data collected in a previous study (Booker et al., 2018); **(B)** crude drug of HP; **(C)** plot comparing crude drug material samples and marketed products.

The NMR data obtained from all the 86 samples was plotted against the data collected by Booker et al. (2018) on marketed HP products, excluding products consisting of extracts and/or combination with other plants. The PCA score plot shows that a few marketed products fall far away from the central cluster (Figure 10). Due to higher variability found among marketed products, the differences between the crude drug samples disappear. The different chemical fingerprints found in the *materia prima* represent the natural chemovariability, (especially the presence of avicularin) which, however, is minimal compared to the differences found in the finished products. The natural variability cannot explain the marketed products variability. Therefore, the reasons behind it need to be found elsewhere. This demonstrates that unregulated products’ significant variation in composition is very heavily influenced by the various processing techniques of the *materia prima*, i.e., the differences in the value chains of these products. This highlights the importance and necessity for a carefully managed and well controlled value chain from the primary material to the finished products.

### Comparison of NMR-PCA and HPTLC

**FIGURE 11:**
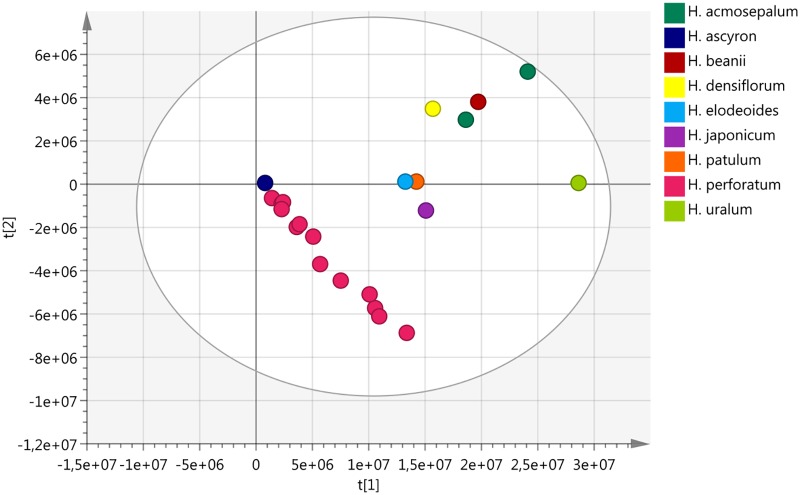
Score plot comparing different *Hypericum* species. *Hypericum perforatum* (pink) is easily distinguishable from the other species, except from *H. ascyron*. The data was acquired using NMR (500 MHz).

Overall, NMR-PCA was able to detect major differences between samples, but has not been useful to discern the much more limited differences between the samples of the *materia prima*. Of course, it is more affected by total composition than a single compound’s variations. It is a useful method for identification of trends and differences between different species, as exemplified in Figure 11, and makes evaluation of the results obtained from large pools of samples easier as it provides a general overview.

HPTLC unveils specific chemical differences. The combination of these two methods has helped an all-round evaluation of the chemical profile and differences existing among the HP available in nature.

## Conclusion

This study demonstrates that the view of ‘Chinese HP’ containing some unique marker substances cannot be substantiated. The HPTLC profiles have highlighted how the Chinese samples and some of the Spanish samples both contain avicularin. At the same time the Chinese samples carry some extra differences that distinguish them from the Spanish avicularin-containing ones. According to the *Hypericum* monographs (Robson, 2002), the distribution of subspecies *perforatum* and *veronense* overlaps in Mediterranean Europe, with minor morphological differences serving as diagnostic markers. On the other hand subspecies *chinense* is quite isolated geographically. As a consequence, it is possible that these detected anomalies, when compared to the EP standard, represent chemotypes characteristic for specific geographical regions. Our samples could not be clearly assigned to these subspecies Moreover, this study demonstrates that rutin, though present in the EP standard and found in all the marketed products analyzed previously (Booker et al., 2018), is not necessarily found in the *materia prima*. The hypericins content was not always directly correlated to the overall flavonoid concentration. It was found to be low in commercial material, either due to higher content of woody material, or unknown age of the sample.

It is vital that there is a standard reference representative of good quality crude drug material taking into consideration the natural chemical variability. Pharmacopeial monographs should include a description of such variable characteristics. In the case of HP, this could either result in accepting *H. perforatum* ssp. *chinense* as a source of drug material if it complies with the other requirements or a new definition of what material is acceptable from a pharmacopeial perspective.

This study for the first time compares a large collection of crude material as a group and also with marketed products, establishing that in the case of HP the naturally occurring chemical differences are not responsible for the poor quality found in the finished commercial products. There is no way of establishing which chemotype has been traditionally used and substantiating that one chemotype is more appropriate than the others. Therefore, these natural differences should not be of major concern. However, in this study all samples were processed using a standard procedure, which is clearly not the case within industry, resulting in inevitable quality variations.

The results regarding the processed material, on the other hand, have highlighted how acquiring material that has been sourced along poorly managed value chains constitutes a concern that needs to be considered and resolved. In such cases the identity, provenance, collection practices, storage conditions and length of storage are unknown and could lead to poor quality material. This strengthens the importance of minimizing the role of middlemen, who lack the knowledge of how to ascertain good quality, operating between growers/collectors and manufacturers.

This study’s findings show the importance of comprehensive investigation and knowledge about crude materials as the foundations for the delivery of quality herbal products on the market. Outreach activities need to target collectors, growers and producers to guarantee that the fundamental steps of cultivating, collecting or acquiring good/acceptable quality material is carried out correctly. If the crude material’s natural variation is known the final product’s quality will be better defined and more predictable.

## Author Contributions

FS, AB, and MH contributed to the conception and design of the study. FS gathered the samples and prepared voucher specimens. FS and KL analyzed the samples. FS analyzed the data and drafted the manuscript. All authors contributed to manuscript revision, read and approved the submitted version.

## Conflict of Interest Statement

The authors declare that the research was conducted in the absence of any commercial or financial relationships that could be construed as a potential conflict of interest.
