# Supplementary figures and images for: St. John’s Wort (Hypericum perforatum) Products – How Variable Is the Primary Material?
Source: Front Plant Sci. 2019 Jan 24;9:1973. doi: 10.3389/fpls.2018.01973 (PMC6357942; doi:10.3389/fpls.2018.01973)

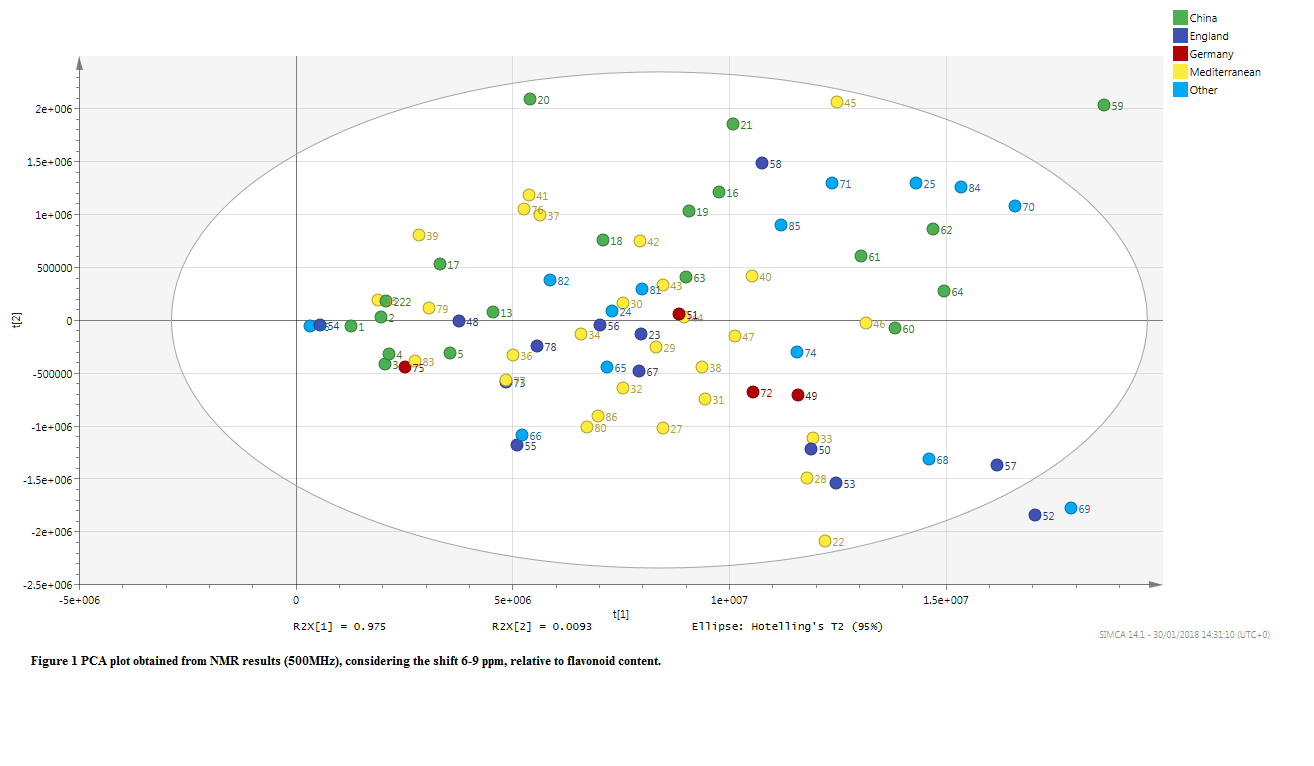

Supplement: Supplementary file 1 [file Image_1.TIF]

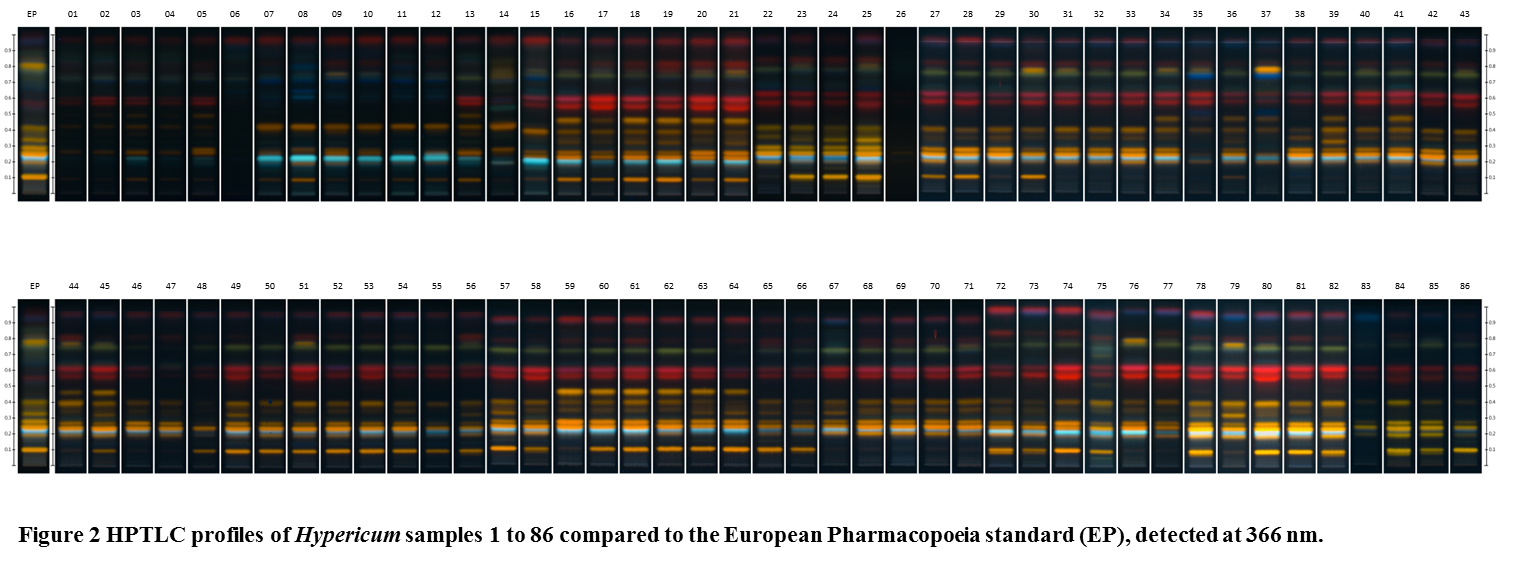

Supplement: Supplementary file 2 [file Image_2.TIF]
